# Improvement of rural soil properties and states by biomass carbon under the concept of sustainability: A research progress

**DOI:** 10.3389/fchem.2022.1078170

**Published:** 2022-11-29

**Authors:** Yuchi Yang

**Affiliations:** College of Architecture and Urban Planning, Tongji University, Shanghai, China

**Keywords:** biomass carbon, soil properties and states improvement, soil pollution, rural sustainable development, research progress

## Abstract

Biomass carbon is a highly aromatic carbonaceous solid obtained by thermochemical reaction of biomass raw materials. It is frequently used in the research and application of soil properties and states improvement. Biomass carbon has abundant porous structure, high specific surface area and surface functional groups. After being applied to the soil, it has a significant impact on manipulating the physichemical properties of the soil, enhancing the microbial environment and remediating soil pollutants, which is conducive to the resource utilization of agricultural wastes and the long-term preservation of the environment. Based on 328 moderately to highly relevant literatures on biomass carbon and rural soil property improvement since 2010, this paper reviewed the contemporary research progress of biomass carbon application in soil property improvements utilizing the concept of sustainable development. In order to provide beneficial illumination for the complete implementation of biomass carbon in improving rural soil properties, this paper primarily evaluated the principle as well as mechanism of promoting sustainable soil properties. It tends to prospect the application and development aspirations of biomass carbon in soil ecological restoration, crop growth, development.

## 1 Introduction

In recent years, the research on the application of biomass carbon in soil properties and states improvement has attracted much attention from the academic community. Recent increases in industrialization and urbanization have resulted in significant CO_2_ emissions, global warming, sewage and other pollutants. As more than just a result, enormous rural regions have unavoidably become the emission receptors for numerous pollutants. Rural soil has become the first place to absorb some heavy metals and toxic chemical elements, so point pollution and non-point source pollution flowing one after another. The rural soil ecological environment has become increasingly worse as a result, which has aroused widespread concern and high attention from the government and academics. Over the years, rural soil has been carrying the important mission of increasing biomass carbon production, absorbing CO_2_ and other carbon containing substances. Improvement of rural soil properties, research and application of sustainable development of soil ecosystems have recently become hot topics in the academic community due to the crisis of heavy metals, toxic chemical element accumulation, as well as changes in soil physichemical structure. The biomass carbon rich in organic carbon has naturally entered the public’s consciousness due to its effective soil improvement performance.

The Amazon basin is where American Indians first applied black carbon to soil to produce fertile black soil known as “Terra Preta” and this is where the phrase “biomass carbon” first appeared (Goldberg, 1985; Lehmann et al., 2003; Lehmann et al., 2008). According to Jiang et al. (2017) and Kong et al. (2015), biomass carbon is the carbonaceous solid product of the high-temperature pyrolysis and carbonization from organic biomass materials including wood, straw, fruit shells in an atmosphere with little to no oxygen. At a relatively modest preparation temperature (<700°C), biomass carbon might well be pyrolyzed to produce valuable carbon-containing compounds (Ahmad et al., 2014; Wang et al., 2022). The characteristics of biomass carbon include high pH, adsorption capacity, abundant nutritional content, large specific surface area, pore structure, stable physichemical properties, and abundant surface functional groups, etc.It may enhance soil, promote soil fertility, encourage crop development and increase soil nitrogen on the impact of chemical circulation because of its unique physichemical properties (Wang et al., 2019; He et al., 2021). High pH, nutritional content, pollutant adsorption ability, rich pore structure, large specific surface area, stable physichemical properties, and a richness of surface functional groups are all features of biomass carbon. When applied to farmland, its unique physical and chemical characteristics can enhance soil, increase soil fertility, encourage crop development, and influence the chemical cycle of soil nitrogen (Wang et al., 2019; Raza et al., 2022).

According to research, biomass carbon improves soil’s physical structure and physichemical properties, which can be utilized as an organic remediation agent in soil systems for carbon sequestration and emission reduction (Wang et al., 2019; Chen F et al., 2022), making it a suitable substance for water and soil remediation (Beesley et al., 2011; Dey et al., 2014; Wu, 2020). According to Spokas et al. (2012), enhancing soil fertility and carbon sequestration, recovering reclaimed land and lowering greenhouse gas emissions from farms are all potential applications for biomass carbon in terrestrial ecosystems (Harter et al., 2014; Troy et al., 2013; Cayuela et al., 2014). Research by academics indicates that biomass carbon can effectively promote the improvement of agricultural soil, the growth and development of crops (Table 1). The expense of preparation is also significantly lower than that of activated carbon, which mostly consumes wood and coal as raw materials. At the same time, its preparation materials originate from a wide range of sources. The application of biomass carbon to enhance soil characteristics will definitely become the primary concern of upcoming researches as a consequence of the progressively severe soil pollution situation.

**TABLE 1:** Research Frontiers of biomass carbon application in soil improvement.

Research topics	Research contents	Researchers	Key words
Biochars’ influence on soil reclaimarion	Research status of biochar for soil improvement and outstanding advantages of carbonization technology	Wang et al. (2014)	biochar; soil reclaimarion; soil ecosystem
Slow-release property and soil remediation mechanism of biochar-based fertilizers	Slow release mechanism and influencing factors of biomass carbon based fertilizer	Zhao et al. (2021)	biochar-based fertilizers; slow-release property; soil improvement
Biochar on transport of inorganic pollutants in soil	the basic characteristics, carbon content and structure of biomass carbon are affected by the properties of source materials	Zhang et al. (2021)	Biochar; adsorbent; Heavy metals; Soil; repair
Soil Physiochemical Properties and Nitrogen Transformation	Changes and response mechanism of nitrogen cycle, nitrogen fixation reaction, ammonification reaction and nitrification reaction driven by soil microorganism	Wang et al. (2019)	Soil physical properties; Soil chemical properties; Microorganism
Effect of biochar on soil physical characteristics improvement	Effect of biochar addition on soil physical characteristics, the best application rate of biochar	Deng et al. (2020)	land consolidation; fertility betterment
Effects of biochar on remediation of heavy metal containeated soil	Application of biochar in remediation of heavy metal contaminated soil	Yang et al. (2020)	Soil heavy metal; mechanism; remediation effect
Remediation of As and Cd contamination by calcium-based magnetic biochar	Solution to the problem of remediation of As and Cd contaminated soil	Wu (2020)	calcium-based magnetic biochar; Cd As co-contamination; bioavailability; paddy soil
Effects of biochar on remediation of heavy metal containeated soil	Application of biochar in remediation of heavy metal contaminated soil	Yang et al. 2020	Soil heavy metal; mechanism; remediation effect
Biochar Behavior in Soil Environment	Behavior of biochar in soil environment and effects of biochar improvement on soilphysihemical properties and crop growth	Zhang et al. (2021)	biochar; soil; environmental behavior; pollutants
Heavy metal contaminated soil using modified biochar	Preparation and properties of biochar and effects of different modification methods on the structural characteristics of biochar	Zhang et al. (2021)	Soil, heavy metals; modified biochar; adsorption
Biochar remediation of petroleum contaminated soil	Physicochemical properties of biochar and biochar remediation of petroleum contaminated soil	Song et al. (2021)	petroleum pollution; remediation technology; soil
Biochar from constructed wetland biomass waste	Characteristics of wetland plant derived biochar, and its utilization in soil improvement, carbon sequestration	Cui et al. (2022)	Aquatic plant Carbon sequestration Sorption Soil improvement
Characteristics of biochar and its effects and mechanism on soil properties	The role of biochar addition in improving soil structure, soil fertility, adjusting soil pH, repairing contaminated soil	Chen F et al. (2022)	biochar; soil properties; influence mechanism; remediation
Effects of biochar addition on nutrient levels and its components in dry farmland soils	The application of biomass carbon in soil improvement and fertility improvement in dry farming areas of the Loess Plateau	Pan et al. (2022)	Loess Plateau; soil nutrient; organic carbon fraction; humic substance

This paper is based on the concept of sustainable development and does use the literature research method. It typically analyzes 328 highly relevant literatures on the topics of biomass carbon and rural soil property improvement since 2010, including 181 Chinese journal literatures in CNKI database and 147 English review journal literatures in WOS database. The production of raw materials, fundamental characteristics, preparation factors, principle and mechanism of biomass carbon to improve rural soil properties are almost all reviewed and analyzed in this paper. The findings are summarized in Table 1 in conjunction with the great potential applications and future development direction of biomass carbon in soil ecological restoration, crop growth and development, research development trend.

## 2 Summary of research progress

The scholars’ research on the improvement of rural soil properties by biomass carbon mainly concentrated on the production of raw materials, basic properties, influencing factors of preparation, the principle and mechanism of biomass carbon to improve rural soil properties and states by sorting out the results of the existing internal and external research.

### 2.1 Raw materials and basic properties of biomass carbon

There are a wide range of raw materials for biomass carbon preparation, such as straw, manure, grass, wood, etc. (Jiang et al., 2017; Liu et al., 2009; Cantrell et al., 2012). Generally, they can be divided into traditional (such as agricultural, forestry wastes and urban wastes) and non-traditional biomass carbon (Zhang et al., 2021; Hossain et al., 2011). There are apparent differences in composition, structure and element types, etc. (Rego et al., 2019; Cao et al., 2022). The physicochemical properties of biomass carbons produced from different raw materials or under different preparation environmental conditions have significant differences and diversity (Aller, 2016), so the impact on crop growth is also very different (Liu et al., 2013; Jeffery et al., 2011). The chemical effects of the same kind of biomass carbons at different temperatures are also different. The differences in specific surface area, cation exchange capacity, the proportions and contents of biomass carbon with different degrees of dissolution of biomass carbon prepared at the same time show significant differences in physical and chemical properties.

Biomass carbon is an important measure for the resource utilization of soil biomass waste and the realization of farmland carbon management. Biomass carbon contains a large number of nutrient elements such as N, P, and K, as well as medium and micronutrient elements such as Ca, S, Fe, and Si, which are used to reduce pests, diseases and ensure the normal growth of soil crops (Zhang, 2017). Researches have shown that biomass carbon is mainly composed of aromatic hydrocarbons, elemental carbon or carbon combinations with graphite-like structure (Chen et al., 2013). Its surface physical and chemical characteristics are of great significance for its potential production and application, which are mainly affected by the temperature of the thermal reaction and raw materials (Chen Y et al., 2022).

Biomass carbon is a solid substance with rich carbon content generated from the pyrolysis of agricultural wastes such as straw (Zhang, 2017). Its carbon fixation and emission reduction effect in farmland soil are mainly due to its high carbon content and highly stable structure (Keiluweit and Johnson 2010). The characterization of biomass carbon is of great significance for its potential applications. Its physical and chemical properties are mainly affected by the thermal treatment temperature and raw materials. Biomass carbon applied to soil can increase soil carbon storage and slow down the carbon cycle process of terrestrial ecosystem (Lehmann et al., 2006; Lehmann et al., 2008; Weng et al., 2017; Zhang, 2017).

### 2.2 Factors affecting the preparation of biomass carbon

Biomass carbon is rich in organic carbon, which is often compared with activated carbon. Activated carbon is mainly made from coal, wood and other materials through high-temperature carbonization reaction, activation and condensation by various preparation methods (Wu et al., 2014; Ahmed et al., 2019). The preparation of raw materials is relatively simple and the source is relatively limited. Biomass carbon is prepared by pyrolysis reaction at low temperature. Its raw materials come from a wide range of sources, such as cheap and rich traditional agricultural wastes (Zhou et al., 2021) and non-traditional biomass (Inyang et al., 2016), such as straw, fruit shells, peanut shells, walnut shells, poultry manure, etc. The preparation cost of biomass carbon is significantly lower than that of activated carbon.

Biomass carbon is a carbon rich solid mixture formed by pyrolysis of biomass wastes such as straw under the condition of oxygen limitation, with an obvious aromatic structure (Raveendran et al.,1995; Brodowski et al., 2005). The constituent elements of biomass carbon are closely related to the preparation temperature in the carbon production process. Specifically, with the increase of carbonization reaction temperature within a certain controllable range, the hydrogen and oxygen content decreases while the carbon and ash contents increase significantly, (Schmidt and Noack 2000; Chen et al., 2013). It is mainly used for recycling agricultural and forestry waste resources or improving farmland soil properties (Liu et al., 2020; Zhang et al., 2021).

### 2.3 Principle and mechanism of improving rural soil properties and states by biomass carbon

#### 2.3.1 Effects of biomass carbon on soil physicochemical properties

The improvement of soil properties by biomass carbon has widely aroused extensive research in academia. The principle of improving soil physical and chemical properties is mainly to affect the soil nitrogen cycle, promote soil carbon sequestration and emission reduction, reduce soil nutrient loss, affect the distribution of soil components and other direct or indirect ways of action. Due to its high pH value, loose porous structure, available carbon nutrient content, biomass carbon plays an important role in mitigating soil acidification, reducing nutrient loss, promoting carbon fixation and emission reduction (Ogura and Date 2016; Ali et al., 2017). Most of the existing researches focus on the overall changes of soil caused by the uniform mixing of biomass carbon and soil, ignoring the spatial heterogeneity and variability of the impact of biomass carbon on the distribution of soil components (Yu and Meng 2019; Wang et al., 2019). Akhtar et al. (2014) finds that biomass carbon is beneficial from improving chlorophyll content, photosynthesis rate (Pn), stomatal conductance (Gs), relative water content (Rwc) and photosynthesis efficiency of tomato leaves. Shafaqat et al. (2017) reveals that the physical and biological characteristics of soil strengthened by biomass carbon under drought conditions improved water conservation capacity, regulated stomatal conductance, plant hormone content, specifically decreased Na^+^, K^+^ absorption of plants. The surface cationic adsorption capacity of biomass carbon can improve the soil cationic exchange capacity. Wang et al. (2019) reveals the different porosity and structure of different soil components are important reasons for the variation of soil water content, rich porous structure, large specific surface area (SSA) and particle mechanical strength could affect the soil water permeability. Biomass carbon is a stable inert substance, which mainly depends on its highly carbonized and aromatic molecular structure (Yu and Meng 2019; Yu, 2021). However, instability phenomena such as aging and decomposition of biomass carbon may occur as time goes by.

Biomass carbon can improve soil by affecting nitrogen transformation. The application of biomass carbon to soil changes the N element cycle, adsorbs and retains N element to a greater extent through its porous characteristics or affects the microbial environment (biodiversity, richness and vitality) in the process of N element cycle. It cannot directly increase the content of N element in soil and mineral N element which are conducive to crop growth and development. Instead, it affects the cycling process of N elements (Wu et al., 2014). Asada et al. (2002) finds that the higher temperature of biomass carbon preparation, the lower number of acid functional groups attached to the surface, and the ability to react to NH_3_ or NH_4_^+^ will be weakened. At the same time, the addition of biomass carbon to the soil will increase the abundance of ammonia oxidizing bacteria, promote the conversion of NH_4_^+^ into NO_3_^−^ in soil (Nelissen et al., 2012), or adsorb phenolic compounds that inhibit nitrification indirectly promoting nitrification (Deluca et al., 2006). Taghizadeh Toosi et al. (2012) focuses on the isotope labeling experiment of N element, the results shows that N element are stable and non-volatile in air, and it could be used by plants after being applied to soil, which reduces the loss of N elements and improves the utilization efficiency. Biomass carbon actually enhanced the nitrogen fixation capacity of soil and enhanced the nitrogen cycle process in soil.

#### 2.3.2 Effects of biomass carbon on soil microbial environment

Biomass carbon improves the soil microecosystem. Its principle is to indirectly promote crop governors by optimizing microbial growth conditions, increasing microbial content, enriching microbial community structure or affecting soil physichemical properties. The uniform and dense pores of biomass carbon can be retained in the soil and form a large number of micropores, which provides a suitable material carrier for the reproduction of microorganisms and the enrichment of community structure. Within a certain range, with the increase of biomass carbon application rate, the number and activity of soil microorganisms significantly increased (Steiner et al., 2007; Simone et al., 2009). Microbial growth requires certain water and temperature conditions, such as drought stress conditions that have a negative impact on crop growth and nutrient absorption (Robertso and Thorbum 2006). These microorganisms can adapt to environmental changes and produce enzymes that decompose soil exogenous substances to promote degradation or transformation of pollutants (Song et al., 2021).

The effects of biomass carbon on soil physichemical properties and states can be divided into direct effects on the growth and development of microorganisms or indirect enhancement of the microbial carrier material richness of physichemical reactions. The influence of biomass carbon on the physichemical properties and states of soil will directly affect the growth and development of microorganisms, which are also important catalysts for soil physichemical reactions and complement to each other. Yu (2021) researches the migration and distribution of biomass carbon in soil, carbon effect, soil microbial diversity and community structure characteristics, nitrogen transformation functional microorganisms in the intercarboniferous microdomain. Yu (2021) researches the effects of biomass carbon on the chemical properties, microbial richness, community structure, soil fertility, crop growth and nitrification of acidified soil, effects on nitrification of acidified soil. Zhang (2017) researches the impact of biomass carbon on agricultural crop productivity, land carbon fixation, emission reduction in different ecosystems, and the impact of biomass carbon application on soil nutrient element rotation (turnover of P, C, and N elements). Zhao et al. (2021) finds that the effect of biomass carbon on crop productivity i significantly different under different soil conditions.

#### 2.3.3 Ecological remediation effect of biomass carbon on soil pollution

Biomass carbon can be used as a functional adsorption material and mixed into soil as a remediation agent. There are many articles on remediation of petroleum soil pollution and heavy metal pollution (Zhang et al., 2021; Erin et al., 2017; El Rasafi and Haddioui 2020). The pollutants are mainly removed through chemical reaction and physical adsorption. Methods used for soil pollution remediation include chemical precipitation, ion exchange, redox and adsorption methods (Xu et al., 2020; Zhang et al., 2021; Wang et al., 2020). Compared with other methods, adsorption method is widely used due to its simple operation, high efficiency, low cost, etc. The research of Bird et al. (1999) and Dai et al. (2005) reveals that under the long-term effect, biomass carbon can physically migrate or decompose to a certain extent, redistribute in the vertical direction of soil and better adsorb pollutants. Xiao et al. (2021) suggest that wetland plant biomass carbon has strong adsorption capacity for various inorganic, organic pollutants and significant effect on soil improvement. Kambo et al. (2015) researches and recognizes the physichemical properties of plant biomass carbon, such as high water content, low calorific value, high volatile components which greatly limits its further application in soil remediation.

The potential toxic substances in soil such as heavy metal ions and polycyclic aromatic hydrocarbons are important material to which need to be paid attention. Biomass carbon can enhance the ability of soil microorganisms to metabolize organic matter, complete the overall adsorption process in order to realize the interaction of all aspects of remediation. At the same time, pollutants may be degraded autonomously in soil as time goes by which also affects the growth and development of soil microorganisms.

## 3 Research conclusion and prospects

**FIGURE 1:**
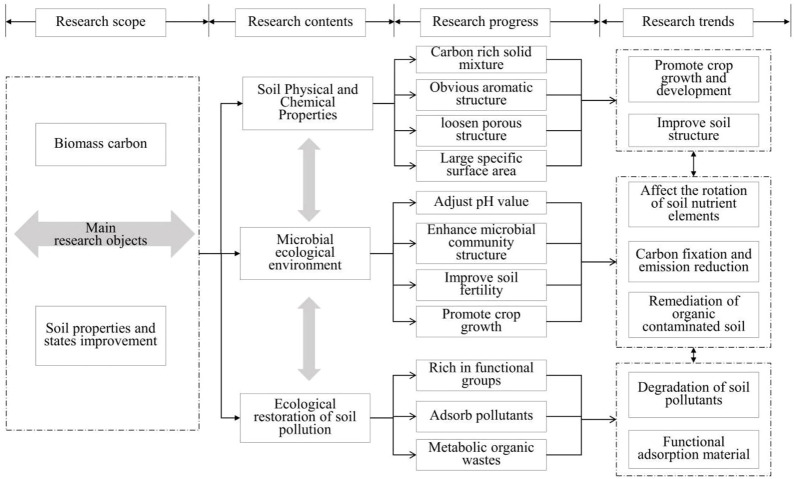
Research progress on improving rural soil properties by biomass carbon.

In recent years, biomass carbon has become a soil improvement material of great concern. Meanwhile, the widespread application of biomass energy will contribute to a reduction in the consumption of fossil fuels and environmental pollution. It does have valuable characteristics such a potent ability for adsorption, simple preparation requirements, low pollution risk and sustainable utility. The resource utilization of agricultural production wastes can be accomplished through the production from several forms of biomass carbon. Regarding soil improvement and restoration, current research primarily concentrates on improving the physichemical characteristics of rural soil, increasing soil fertility and enriching soil microbial communities. The preparation of different types of biomass carbon can realize the resource utilization of agricultural production wastes. Existing researches mainly focus on the improvement of physichemical properties of rural soil, the improvement of soil fertility and the enrichment of soil microbial communities to achieve soil improvement and restoration. Despite the existence of multiple breakthroughs when field experiments are combined with biomass carbon, there are still many issues that need to be resolved before biomass carbon could be extensively used for rural soil improvement, remediation, and establishing standardized approaches. During the long-term application process in actual agricultural production, it is critical to take the environment’s dangers into account as well as the biomass carbon’s appropriate environment. Following is an extensive description of the crucial challenges and emerging trends in research on enhancing rural soil properties by biomass carbon based on an analysis of academics’ research results and contemporary development demands (Figure 1).

### 3.1 Research defects

Biomass carbon can not only improve rural soil environment, but also be used as slow release pesticide and growth fertilizer in agricultural production process. Existing researches are deficient in the following aspects. The existing researches are insufficient in the following aspects: 1) How to standardize the combined application of biomass carbon with different content, type, proportion to cope with specific soil improvement process and take enduring effect quickly. 2) How does the biomass carbon cope with the soil improvement process under different climate, temperature, humidity conditions, realize the long-term carbon fixation and emission reduction of farmland. 3) During the standardized preparation of biomass carbon, we should pay attention to the process conditions, original sources and relatively stable components to minimize the potential environmental risks of biomass carbon as much as possible. 4) When biomass carbon was applied to soil, unknown organic matter would be released during the aging process, and its long-term harmfulness to the environment needs further attention in a long time. 5) The biomass carbon to soil pollutants should be repair for single contaminant to compound pollutants, from a single biomass carbon to a variety of biomass carbon synergy mechanism researches and an extensive experiment. 6) Biomass carbon can not only be used as soil improvement and pollution remediation, but also as slow release pesticide and fertilizer application in the fields together. 7) From the macroscopic action process of biomass carbon to the microscopic in-depth research process of biomass carbon. To research the free radicals on the surface of biomass carbon material, the functional structure of molecules, the efficiency of action, effectiveness, etc. At the same time, it should be shifted from the research of short-term effects to the comprehensive application and in-depth tracking research of long-term environmental benefits. 8) Biomass carbon has a broad development prospect in the future, 1 combination is further broaden its applications such as in the village planning, such as in village planning, rural community construction, regional carbon reduction and emission reduction, and rural revitalization. So as to continue to expand its research and attention to environmental protection and economic applications.

### 3.2 Research prospects

The focus as well as objectives of economic and social development in the new era are always to prevent global warming, reduce CO_2_ and other greenhouse gas emissions, develop circular and sustainable agriculture, construct green and low-carbon communities, implement rural revitalization strategies, encourage sustainable land spatial patterns, and create ecological civilizations. The capacity to guarantee food security and maintain sustainable development of rural soil are both important goals. Additional researches will concentrate on the potential of biomass carbon for crop growth and development as well as its application to the ecological rehabilitation of rural soil. How to batch and modularize the preparation of different kinds of biomass carbon, and actively invest in the process of rural soil environmental restoration. How to determine appropriate means and pathway of improving soil with biomass carbon according to different types of pollutants, so as to maintain a more stable remediation niche. In the process of further research and practice, more stable application of multiple biomass carbon in rural soil remediation such as content needs to be emphatically considered in the future application.

#### 3.2.1 Methods and efficiency of biomass carbon for ecological restoration of rural soil

Biomass carbon remediation of organically polluted soil is mainly achieved through adsorption, and the adsorption mechanism mainly includes three ways such as distribution, surface adsorption, pore interception. Due to the increase in the types and quantities of organic pollutants in recent years, the remediation process of soil ecological restoration has become more complex, and the adsorption of pollutants is more difficult. So it is necessary to pay attention to the joint application of multiple adsorption methods. Single biomass carbon is difficult to effectively solve the problem of soil ecological restoration, which needs to be used together with other remediation measures such as organic fertilizer. According to the systematic principle, different types, dosage and ratio of biomass carbon combined use, application conditions, environment and other relevant reference standards for future applications need to be referred to in the future. Biomass carbon which has emerged in recent years shows great adsorption performance for heavy metals due to its rich functional groups and high specific surface area, which can effectively reduce the bioavailability and mobility of heavy metals in soil. For different types of pollutants, how to determine the most appropriate way to improve biomass carbon needs further research, and the adsorption efficiency and action intensity of different types of biomass carbon need to be concerned. It is necessary to turn short-term laboratory experiments into long-term integrated field experiments, systematically consider the remediation and application effects of biomass carbon in different soil types, establish long-term location tracking experiments or isotope experiments. Focusing on the advantages of modified biomass carbon, we need to be cautious about its negative effects in the repair process. During the repair process, potential hazardous substances may be released and there is a risk of secondary pollution at the same time. Under the concept of sustainable development, it is necessary to fully consider the safety of biomass carbon and give full attention to the biomass charcoal degradation ability of soil pollutants, which is of great practical significance to improve the functional characteristics of biomass carbon.

#### 3.2.2 Effects of biomass carbon on crop growth and yield

Biomass carbon affects plant growth and photosynthetic efficiency by improving soil phychemical properties and increasing plant water content. The influence of biomass carbon on soil water retention rate is different due to the content, source, application amount of biomass carbon and other pertinent factors. The activity and retention efficiency of biomass carbon are affected by the preparation method and process. The external surface of biomass carbon has oxidation capacity and can adsorb more metal cations such as Al^3+^, H^+^. It contains Ca^2+^, K^+^, Mg^2+^, Na^+^ and other salt ions, which can improve the exchange capacity and frequency of cations in soil. The research found that the effects of biomass carbon on crop growth, development and yield are mainly focused on as follows: Biomass carbon by improving soil phychemical properties and states, increase water content of plants to affect plant growth, photosynthesis efficiency. Biomass carbon to soil water retention effect because of factors such as biomass carbon content, source and find the differences. The influence of biomass carbon on soil water retention rate is different due to the content, source and application amount of biomass carbon. The activity and retention efficiency of biomass carbon are affected by the preparation method and process. The outer surface of biomass carbon has oxidation ability, which can absorb more metal cations such as Al^3+^ and H^+^. It contains Ca^2+^, K^+^, Mg^2+^, Na^+^ and other salt ions, which can improve the exchange capacity and frequency of cations in soil. This research actually finds that the effects of biomass carbon on crop growth, development and yield are mainly concerned as follows: 1) The multi-microporous structure of biomass carbon provides the content of bacteria in the soil which is conducive to growth and enriches the growth environment. 2) Enhancing soil available nutrients content such as porosity, water retention, pH, salinity, electrical conductivity (EC), cation exchange capacity (CEC) and other physicochemical properties. 3) Biomass carbon contains rich trace elements such as N, P, K which can effectively exchange with metal cations in the soil to provide more growth elements, etc. 4) Impact on soil carbon cycle, carbon sequestration and emission reduction: carbon sequestration and storage in soil can improve soil structure, nutrient content and reduce greenhouse gas emissions such as CO_2_. 5) To promote the growth of seed germination plants and increase crop yield. It is noteworthy that the porous structure and alkalinity of biomass carbon play an important role in improving the properties of acid soil and increasing crop yield. However, biomass carbon with high soil pH value will lead to poor crop growth. Tar and resin which are generated in the preparation process will inhibit crop growth. In further research, the focus should be on the pollution recovery of large-scale application of biomass carbon in the ecosystem, the adsorption and transport of natural organic matter on biomass carbon. How to maintain the same growth efficiency and improve crop yield in soil crops with different environmental conditions such as pH, temperature, etc.

In summary, in order to better comprehend how biomass carbon affects soil quality, several large-scale and long-term field application research involving biomass carbon should be undertaken in the future. It must emphasize the breadth, depth, or application of development. Pay sincere attention to how it is used in the processes of improving the soil and eradicating pollution, as well as the long-term, short-term effects (both positive and negative effects). The latest research results will be widely used in crop practice.
